# Supplementary material for: Impact of the internet use on informal workers’ wages: Evidence from China
Source: PLoS One. 2023 May 19;18(5):e0285973. doi: 10.1371/journal.pone.0285973 (PMC10198487; doi:10.1371/journal.pone.0285973)
Supplement: Attachment — Submitted filename: Response to Reviewers.docx [file pone.0285973.s002.docx]

**Response to Reviewers**

Thank you to the Academic Editor and the Reviewers for the valuable suggestions provided for our manuscript. We have made every effort to revise the paper according to the suggestions. Below is a brief response to the suggestions provided:

**Reviewer #1:**

**1. In the introduction section- the authors need to develop the theoretical/conceptual link between internet use and wages and need for this study. I know authors did talk on this in the second paragraph but in my view it’s not enough. Adding more insights on this will not only make a strong theoretical background of the paper but also give justification for need to undertake this kind of analysis.**

Answer: We have made modifications to the introduction section, highlighting the theoretical/conceptual link between Internet use and wages, as well as the significance of this study.

**2. On page 10 - interpretation of results. Check on the interpretation of variable gender. Was the wage variable in logs?**

Answer: Wages are represented in logarithmic form to eliminate the influence of heteroscedasticity.

**3. The authors also claim that there is gender wage discrimination based on the estimated coefficient of 0.397. I do not think you can claim such statements. Because differential of 39.7% could be due to gender related or factors associated with gender that you may have not controlled for in the regression. For instance, being male may be correlated with other productivity characteristics that have not being controlled for.**

Answer: Thank you for your suggestion. We have made modifications accordingly and removed statements such as "claiming the existence of gender wage discrimination". The revised version has been highlighted in the manuscript.

**4. On page 11 – on family size. Authors justify negative impact of family size using ‘Many minor children’. Families in China can only have 2 kids which is normal with respect to replacement level of fertility so justifying this a lot is does not augur well with China’s child policy.**

Answer: We have defined the concept of family size in the previous section, i.e., the number of underage children. We use "number of underage children" to demonstrate the negative impact of family size, only examining the impact of family size on wages from the perspective of labor supply. The larger the family size, the more underage children there have to be taken care of, and a greater amount time is needed to do so. This explains its negative effect on labor supply and overall wage levels.

**5. Avoid using ‘Seen from…” all the time to begin your sentences.**

Answer: Thank you for pointing out this issue. We have carefully reviewed the manuscript and made modifications to phrases such as "Seen from...".

**Reviewer #2:**

1. Is the manuscript technically sound, and do the data support the conclusions?

The paper is well written and it is faithful to the hypotheses. The data has been sourced from a valid provider and sampled appropriately. The authors need to state the motivation for conducting the following research in the introduction section alongisde the hypothesis. Additionally, authors are recommended to ouline the major findings in the introduction for the readers.
Answer: We have made modifications to the introduction section and outlined the major findings.
2. Has the statistical analysis been performed appropriately and rigorously?

All the analysis and tests have been carried out to establish the research hypothesis.

Answer: Thanks for your suggestion. In the manuscript, we have empirically analyzed the impact of Internet use on informal workers' wage by constructing models of baseline regression, endogenous transformation, and treatment effects. We have also verified the robustness of the empirical models with robustness tests and various methods. Finally, we have conducted a heterogeneity analysis to explore the impact of Internet use on informal workers' wages by category.
3. Have the authors made all data underlying the findings in their manuscript fully available?

All the required findings are made available in the findings section. Authors are recommended to make the data available for replication.

Answer: The data used in the manuscript comes from the China Family Panel Studies (CFPS) by the Institute of Social Science Survey (ISSS) of Peking University, aiming to reflect the changes in Chinese society, economy, population, education and health through tracking and collecting data at the levels of individuals, families, and communities, and to provide data for academic research and public policy analysis. It is a national, large-scale, interdisciplinary social tracking survey project, and data sources can be downloaded from http://www.isss.pku.edu.cn/cfps/. The sample data used in the manuscript can be obtained by processing original data according to the following steps: (1) screen out agricultural employment samples; (2) collect samples with weekly working hours of 30–84 hours; (3) retain male samples aged 16–60 and female samples aged 16–55 according to mandatory retirement age in China; (4) remove invalid questionnaires with logical errors, missing key information, and refusal to answer. Meanwhile, the manuscript has taken into account the interference of wage income outliers and therefore performed top and bottom 1% trimming on hourly wage income.

4. Is the manuscript presented in an intelligible fashion and written in standard English?
The manuscript is well presented and clearly written.

Answer: Thanks for your recognition. We will continue to carefully revise the manuscript until it fully meets the requirements of the journal.
